# Neurocognitive screening in patients following SARS-CoV-2 infection: tools for triage

**DOI:** 10.1186/s12883-022-02817-9

**Published:** 2022-07-30

**Authors:** Karen Blackmon, Gregory S. Day, Harry Ross Powers, Wendelyn Bosch, Divya Prabhakaran, Dixie Woolston, Otto Pedraza

**Affiliations:** 1 Department of Psychiatry and Psychology, Mayo Clinic, 4500 San Pablo Road, Jacksonville, FL 32224 USA; 2 Department of Neurology, Mayo Clinic, Jacksonville, FL USA; 3 Division of Infectious Diseases, Mayo Clinic, Jacksonville, FL USA; 4 Center for Individualized Medicine, Mayo Clinic, Jacksonville, FL USA; 5 Department of Psychiatry and Psychology, Mayo Clinic, Phoenix, AZ USA

**Keywords:** COVID-19, Long COVID, Neuroinfectious disease, Neuropsychology, Memory, Post intensive care unit syndrome, Myalgic encephalomyelitis

## Abstract

**Background:**

Cognitive complaints are common in patients recovering from Coronavirus Disease 2019 (COVID-19), yet their etiology is often unclear. We assess factors that contribute to cognitive impairment in ambulatory versus hospitalized patients during the sub-acute stage of recovery.

**Methods:**

In this cross-sectional study, participants were prospectively recruited from a hospital-wide registry. All patients tested positive for SARS-CoV-2 infection using a real-time reverse transcriptase polymerase-chain-reaction assay. Patients ≤ 18 years-of-age and those with a pre-existing major neurocognitive disorder were excluded. Participants completed an extensive neuropsychological questionnaire and a computerized cognitive screen via remote telemedicine platform. Rates of subjective and objective neuropsychological impairment were compared between the ambulatory and hospitalized groups. Factors associated with impairment were explored separately within each group.

**Results:**

A total of 102 patients (76 ambulatory, 26 hospitalized) completed the symptom inventory and neurocognitive tests 24 ± 22 days following laboratory confirmation of SARS-CoV-2 infection. Hospitalized and ambulatory patients self-reported high rates of cognitive impairment (27–40%), without differences between the groups. However, hospitalized patients showed higher rates of objective impairment in visual memory (30% vs. 4%; *p* = 0.001) and psychomotor speed (41% vs. 15%; *p* = 0.008). Objective cognitive test performance was associated with anxiety, depression, fatigue, and pain in the ambulatory but not the hospitalized group.

**Conclusions:**

Focal cognitive deficits are more common in hospitalized than ambulatory patients. Cognitive performance is associated with neuropsychiatric symptoms in ambulatory but not hospitalized patients. Objective neurocognitive measures can provide essential information to inform neurologic triage and should be included as endpoints in clinical trials.

**Supplementary Information:**

The online version contains supplementary material available at 10.1186/s12883-022-02817-9.

## Background

Cognitive complaints are common in patients recovering from Coronavirus Disease 2019 (COVID-19) [1]. In many cases, it may be unclear whether complaints reflect neurologic injury [2] or the combined (and potentially reversible) effects of depression, anxiety, and sleep dysfunction, which are common in recovering patients [3]. Focal neurologic injuries, including ischemic and hemorrhagic strokes, are more common in hospitalized relative to ambulatory patients [2]. Yet, subjectively reported symptoms of brain fog, weakness, fatigue, and myalgia are elevated in ambulatory patients [3], to a higher degree than hospitalized patients in some reports [4]. Different etiological factors likely contribute to neurocognitive complaints in patients with mild versus severe COVID-19, influencing treatment and prognosis. Neuropsychological triage may help disentangle the contributions of focal neurological injuries and non-focal contributions of mood, anxiety, and sleep disorders to neurocognitive deficits in recovering patients.

A prior systematic review concluded that acute illness severity was not predictive of post-acute cognitive outcomes [1]. However, most studies to date have been limited by use of subjective symptom reporting to probe cognitive endpoints [5–8]. Subjective measures are only weakly associated with objective neurocognitive performance in COVID-19 [9]. Among studies that utilized objective measures, most involved hospitalized patients only [9–13], which limits the range of illness severity, or utilized abbreviated screening measures [11, 13, 14], which limits detection of focal (i.e., domain-specific) cognitive effects. When a more extensive neuropsychological assessment battery was administered to patients approximately 7–8 months after infection, hospitalized patients were more likely to show impairment in attention, executive functioning, verbal fluency, and memory than outpatients [15]; however, the relationship between cognitive performance and neuropsychiatric complaints was not evaluated.

In the current study, we prospectively assessed subjective and objective neuropsychological profiles in a mixed cohort of ambulatory and hospitalized patients. Participants were assessed early in recovery from COVID-19 using a remote, multi-domain, computerized cognitive test platform. We hypothesized that hospitalized patients would show a higher incidence of objective cognitive impairment than ambulatory patients and that elevated depression, anxiety, fatigue, and pain would be associated with lower cognitive performance in ambulatory patients.

## Methods

### Participants

Participants were recruited via email from a hospital-wide registry of patients at Mayo Clinic (Jacksonville, Florida). All patients tested positive for severe acute respiratory syndrome from coronavirus 2 (SARS-CoV-2) infection between June 2020 and March 2021. Infection status was determined by a real-time reverse transcriptase polymerase-chain-reaction (RT-PCR) assay from nasopharynx swab specimens. Participants ≤ 18 years-of-age and those with a history of major neurocognitive disorder were excluded. To participate, patients required access to a home desktop or laptop computer for test and survey completion. A small number of participants completed assessments from their hospital room and in those cases, a research laptop was provided. The majority of participants completed study assessments in their homes, with research staff available by phone for assistance.

### Clinical and sociodemographic variables

Patient demographics and health history were extracted from the electronic medical record. The COVID-19 Risk Calculation Score (CRC) [16] and Elixhauser-van Walraven Comorbidity Index (EVCI) [17] were derived for all participants to assess the relationship between pre-existing health comorbidities and hospitalization status. The Covid-19 Disease Severity Score [18] was calculated to characterize the severity of their infection. Participants were contacted by phone if there was insufficient information in the medical chart to characterize their acute infection symptoms and/or health history.

### Neuropsychological assessment

Participants were sent a link via email to complete an extensive neurobehavioral questionnaire (Neuropsych Questionnaire-45) [19] and a computerized cognitive screen (CNS-Vital Signs) [20] from their home or hospital room. Test instructions, practice trials, and validity indices were integrated into the test platform to ensure comprehension of instructions, with research staff available for clarification by phone if needed.

#### Subjective

The Neuropsych Questionnaire-45 [19] surveys subjective complaints of attention (e.g., difficulty concentrating, easily distracted), memory (e.g., forgetful, misplacing items), anxiety (e.g., feeling nervous, tense, worrying too much), depression (e.g., discouraged about the future, little or no interest in things), fatigue (e.g., low energy, weak), sleep (e.g., hard to fall asleep, disturbed sleep), and pain (e.g., back pain, headache, muscle soreness). Domain scores are summed and classified as minimal (0–74), mild (75–149), or moderate to severe (150–300) problems with neuropsychiatric functioning [19].

#### Objective

The CNS-Vital Signs core test battery is comprised of 7 subtests: verbal memory, visual memory, finger tapping (motor speed), symbol digit coding, Stroop (selective attention), shifting attention (set-shifting), and continuous performance test (vigilance/sustained attention). From these, 7 domain scores are extracted (Supplementary Table 1), as described elsewhere [20]. Performance validity indicators are embedded within tests, with cut-off values specified in the CNS-Vital Signs Interpretation Guide [21]. We adjusted domain scores for age based on a normative reference group (mean = 100; SD = 15), which was collected prior to the COVID-19 pandemic [20]. We classified impairment (< 9^th^ percentile) based on the American Academy of Clinical Neuropsychology consensus conference statement on uniform labeling of performance test scores [22].

### Statistical analyses

Sample variables were summarized using descriptive statistics. Subjective and objective neuropsychological measures were evaluated for normalcy using the Shapiro–Wilk test following removal of invalid data. Group means, variance, and proportions were derived and compared between the ambulatory and hospitalized groups using t-tests for continuous data or chi-square and Fisher’s exact test for categorical and binomial data, respectively. Nonparametric (Spearman) correlations were used to probe associations between subjective symptom ratings and objective cognitive performance. Level of significance was set at *p* < 0.05, two-sided, and corrected for multiple comparisons using the Benjamini–Hochberg adjusted false discovery rate [23] of 0.014 for the 7 neurocognitive domains that were tested.

### Data availability

Data not provided in the article and additional information on methods and materials will be shared upon reasonable request from qualified investigators.

## Results

### Sample characteristics

One hundred forty-nine patients consented to study participation. Of these, 102 (76 ambulatory, 26 hospitalized) completed the computerized test battery and symptom questionnaire (68.5%). There were no differences between participants that did and did not complete outcome assessments in age (*p* = 0.15), sex (*p* = 0.72), race (*p* = 0.58), or ethnicity (*p* = 0.47). However, a larger proportion of ambulatory patients did not complete outcome assessments compared with hospitalized patients (*p* = 0.007; Supplementary Table 2). Sociodemographic and clinical characteristics of the cohort that completed outcome assessments are presented in Table 1. There were no differences in the duration of time between laboratory confirmation of COVID-19 infection and assessment between the ambulatory (mean = 23.77 days; SD = 22.01) and hospitalized (mean = 28.76 days; SD = 33.02) groups (t = 0.14; *p* = 0.89). Twenty-six patients were hospitalized due to respiratory complications/pneumonia (*n* = 23), delirium (*n* = 1), syncope (*n* = 1), and history of chronic respiratory disease without acute pneumonia (*n* = 1). The remainder of the sample remained ambulatory without requirement for supplemental oxygen. Anosmia was reported in 9/26 (35%) hospitalized and 13/76 (17%) ambulatory patients (*p* = 0.095). Age, race, CRC score, EVCI score, and a specific history of hypertension, coronary artery disease, diabetes, obesity, and smoking were associated with hospitalization status. Five hospitalized patients had documented mental status changes; none had evidence of seizures or stroke.

**Table 1:** Demographic and clinical characteristics of the cohort

	**Overall (*N* = 102)**	**Ambulatory (*N* = 76)**	**Hospitalized** **(*N* = 26)**	***p-value***
**Age**, mean (SD)	52.21 (14.83)	50.41 (14.52)	57.46 (14.72)	0.04
**Education in years**, mean (SD)	15.78 (2.45)	16.06 (2.37)	14.96 (2.53)	0.06
**Sex**, n (%)				0.49
Males	44 (43%)	31 (41%)	13 (50%)	
Females	58 (57%)	45 (59%)	13 (50%)	
**Race**, n (%)				0.03
Native American	0 (0%)	0 (0%)	0 (0%)	
Asian	5 (5%)	2 (3%)	3 (12%)	
Native Hawaiian	0 (0%)	0 (0%)	0 (0%)	
Black	9 (9%)	4 (5%)	5 (19%)	
White	87 (85%)	69 (91%)	18 (69%)	
More than one race	1 (1%)	1 (1%)	0 (0%)	
**Ethnicity**, n (%)				0.2
Hispanic	8 (8%)	4 (5%)	4 (15%)	
Non-Hispanic	94 (92%)	72 (95%)	22 (85%)	
**The COVID-19 Risk Calculation Score (CRC)**, n (%)				< 0.044
0–2 Points: Low Risk	69 (68%)	56 (74%)	13 (50%)	
3–5 Points: Medium Risk	30 (29%)	19 (25%)	11 (42%)	
> = 6 Points: High Risk	3 (3%)	1 (1.3%)	2 (8%)	
**Elixhauser-van Walraven Comorbidity Index (EVCI)**, mean (SD)	4.60 (7.62)	2.39 (6.35)	11.04 (7.49)	< 0.001
**COVID-19 Severity**, n (%)				< 0.001
Asymptomatic	11 (11%)	11 (15%)	0 (0%)	
Mild – No Hypoxia	67 (65%)	65 (85%)	2 (8%)	
Moderate—Pneumonia	4 (4%)	0 (0%)	4 (15%)	
Severe—Pneumonia	17 (17%)	0 (0%)	17 (65%)	
Critical—ARDS	3 (3%)	0 (0%)	3 (12%)	
Critical—Sepsis	0 (0%)	0 (0%)	0 (0%)	
**Comorbid Hypertension**, n (%)				0.002
no	66 (67%)	55 (75%)	11 (42%)	
yes	33 (33%)	18 (25%)	15 (58%)	
**Comorbid Coronary Artery Disease**, n (%)				0.015
no	86 (87%)	67 (92%)	19 (73%)	
yes	13 (13%)	6 (8%)	7 (27%)	
**Comorbid Diabetes**, n (%)				0.011
no	89 (90%)	69 (95%)	20 (77%)	
yes	10 (10%)	4 (5%)	6 (23%)	
**Comorbid Obesity**, n (%)				0.046
no	87 (88%)	67 (92%)	20 (77%)	
yes	12 (12%)	6 (8%)	6 (23%)	
**Comorbid Chronic Neurologic Disease**, n (%)				0.953
no	95 (96%)	70 (96%)	25 (96%)	
yes	4 (4%)	3 (4%)	1 (4%)	
**Comorbid Chronic Psychiatric Disease**, n (%)				0.309
no	73 (73%)	56 (76%)	17 (65%)	
yes	27 (27%)	18 (24%)	9 (35%)	
**Smoking History**, n (%)				0.04
Never Smoked	72 (71%)	57 (75%)	15 (58%)	
Past or Current Smoker	26 (25%)	15 (20%)	11 (42%)	
Unknown/Refused	4 (4%)	4 (5%)	0 (0%)	

### Subjective neuropsychological complaints

Symptom inventory data were non-normally distributed. Moderate-to-severe problems in attention were reported in 21/73 (29%) ambulatory patients and 9/25 (36%) hospitalized patients, with no difference between the groups (*p* = 0.44). Moderate-to-severe problems in memory were reported in 20/73 (27%) of the ambulatory patients and 10/25 (40%) of the hospitalized patients, with no difference between the groups (*p* = 0.17). Moderate-to-severe problems with anxiety were reported in 12/73 (16%) of the ambulatory patients and 4/25 (16%) of the hospitalized patients, with no difference between the groups (*p* = 0.99). Moderate-to-severe problems with depression were reported in 10/73 (14%) of the ambulatory patients and 3/25 (12%) of the hospitalized patients, with no difference between the groups (*p* = 0.46). Moderate-to-severe problems with fatigue were reported in 33/73 (45%) of the ambulatory patients and 12/25 (48%) of the hospitalized patients, with no difference between the groups (*p* = 0.94). Moderate-to-severe problems with sleep disturbance were reported in 24/73 (33%) of the ambulatory patients and 14/25 (56%) of the hospitalized patients, with no difference between the groups (*p* = 0.12). Finally, moderate-to-severe problems with pain were reported in 18/73 (24%) of the ambulatory patients and 10/25 (40%) of the hospitalized patients, with no difference between the groups (*p* = 0.26).

In ambulatory patients, self-reported problems with attention were strongly correlated with the perceived severity of anxiety (rho = 0.58; *p* < 0.001), depression (rho = 0.65; *p* < 0.001), fatigue (rho = 0.69; *p* < 0.001), sleep dysfunction (rho = 0.54; *p* < 0.001), and pain (rho = 0.39; *p* = 0.001). Self-reported problems with memory were strongly correlated with perceived severity of anxiety (rho = 0.59; *p* < 0.001), depression (rho = 0.61; *p* < 0.001), fatigue (rho = 0.64; *p* < 0.001), sleep dysfunction (rho = 0.48; *p* < 0.001), and pain (rho = 0.44; *p* = 0.001).

**Fig. 1:**
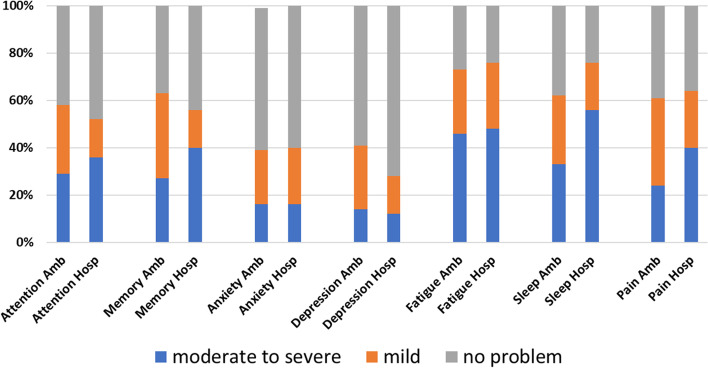
Subjective symptom severity ratings on a self-report inventory. Ambulatory (Amb) patients did not differ in symptom severity ratings from patients who required hospitalization (Hosp) for COVID-19

Within the hospitalized group, self-reported problems with attention were strongly correlated with perceived severity of anxiety (rho = 0.64; *p* < 0.001), fatigue (rho = 0.51; *p* < 0.001) and sleep dysfunction (rho = 0.73; *p* < 0.001) but not depression (rho = 0.38; *p* = 0.07) or pain (rho = 0.17; *p* = 0.49). Self-reported problems with memory were strongly correlated with perceived severity of sleep dysfunction (rho = 0.58; *p* = 0.003) but not anxiety (rho = 0.33; *p* = 0.10), depression (rho = 0.37; *p* = 0.06), fatigue (rho = 0.35; *p* = 0.09), or pain (rho = 0.04; *p* = 0.84; Fig. 1).

### Objective neurocognitive testing

**Fig. 2:**
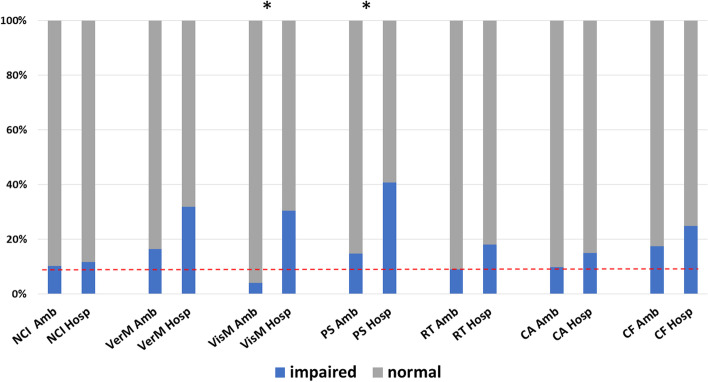
Rates of objective impairment on neurocognitive testing. Patients who were hospitalized (Hosp) for COVID-19 showed higher rates of impairment in visual memory and psychomotor speed compared with patients who remained ambulatory (Amb). Impairment was defined by age-adjusted standardized scores < 9th percentile (red dashed line = 9%). Asterisk indicates *p*-value < 0.05

Neurocognitive performance data were normally distributed. A total of 76/102 (75%) of patients who completed the computerized test battery had sufficiently valid domain scores to derive the overall neurocognitive index (NCI). A total of 8/76 (11%) obtained an NCI score in the impaired range. Rates of overall neurocognitive impairment did not differ between ambulatory [6/59 (10%)] and hospitalized [2/17 (12%)] patients (*p* = 0.85). However, the groups differed in specific cognitive domain performance. The rate of visual memory impairment was higher among hospitalized [7/23 (30%)] than ambulatory [3/73 (4%)] patients (*p* = 0.001). The rate of psychomotor speed impairment was also higher among hospitalized [9/22 (41%)] than ambulatory [11/74 (15%)] patients (*p* = 0.012). Rates of performance impairment for each domain by group are presented in Table 2 and Fig. 2. Mean domain scores for each group are presented in Supplementary Table 3. Ambulatory patients performed within the average range on all 7 neurocognitive test domains. Among hospitalized patients, mean performance on verbal memory, psychomotor speed, and reaction time measures were in the low average range.

**Table 2:** Prevalence of cognitive impairment during recovery from COVID-19

	Impaired (< 9^th^ percentile), n (%)			Odds ratio (95% CI)
	Total (*N* = 102)	Ambulatory (*N* = 76)	Hospitalized (*N* = 26)	Hospitalized vs Ambulatory
Neurocognitive Index	9 (12%)	6 (10%)	3 (17%)	1.18 (0.22–6.44)
Verbal Memory	20 (20%)	12 (16%)	8 (32%)	2.39 (0.84–6.79)
Visual Memory	10 (10%)	3 (4%)	7 (30%)	10.21 (2.38–43.85)
Psychomotor Speed	20 (11%)	11 (15%)	9 (41%)	2.63 (0.82–8.40)
Reaction Time	10 (11%)	6 (9%)	4 (18%)	2.22 (0.56–8.75)
Complex Attention	9 (11%)	6 (10%)	3 (15%)	1.62 (0.37–7.12)
Cognitive Flexibility	16 (19%)	11 (17%)	5 (25%)	1.58 (0.47–5.25)

### Predictors of objective neurocognitive performance

We examined the relationship between demographic/clinical factors associated with disease severity (i.e., hospitalization status) and objective performance on the visual memory and psychomotor speed indices to determine whether they may be contributing to the higher impairment rates among the hospitalized group. Within the combined hospitalized and ambulatory groups, there was no relationship between visual memory impairment and age (t = 0.65, *p* = 0.51), race (χ2 = 2.27, *p* = 0.53), comorbid hypertension (χ2 = 0.31, *p* = 0.58), coronary artery disease (χ2 = 0.15, *p* = 0.70), diabetes (χ2 = 1.37, *p* = 0.24), obesity (χ2 = 0.50, *p* = 0.48), or smoking history (χ2 = 0.72, *p* = 0.70). There was no relationship between psychomotor speed impairment and age (t = 0.51, *p* = 0.61), race (χ2 = 0.35, *p* = 0.95), comorbid hypertension (χ2 = 0.52, *p* = 0.47), coronary artery disease (χ2 = 0.12, *p* = 0.73), or smoking history (χ2 = 1.20, *p* = 0.55). However, reduced psychomotor speed was associated with obesity (χ2 = 4.81, *p* = 0.028) and diabetes (χ2 = 7.57, *p* = 0.006).

Although education level was marginally associated with hospitalization status, we investigated the relationship between years of education and neurocognitive performance, given that it has served as an important predictor in prior studies [10, 12]. Higher education was associated with higher overall NCI (rho = 0.29, *p* = 0.014) and psychomotor speed (rho = 0.22, *p* = 0.04), but not visual memory (rho = 0.16, *p* = 0.14).

**Fig. 3:**
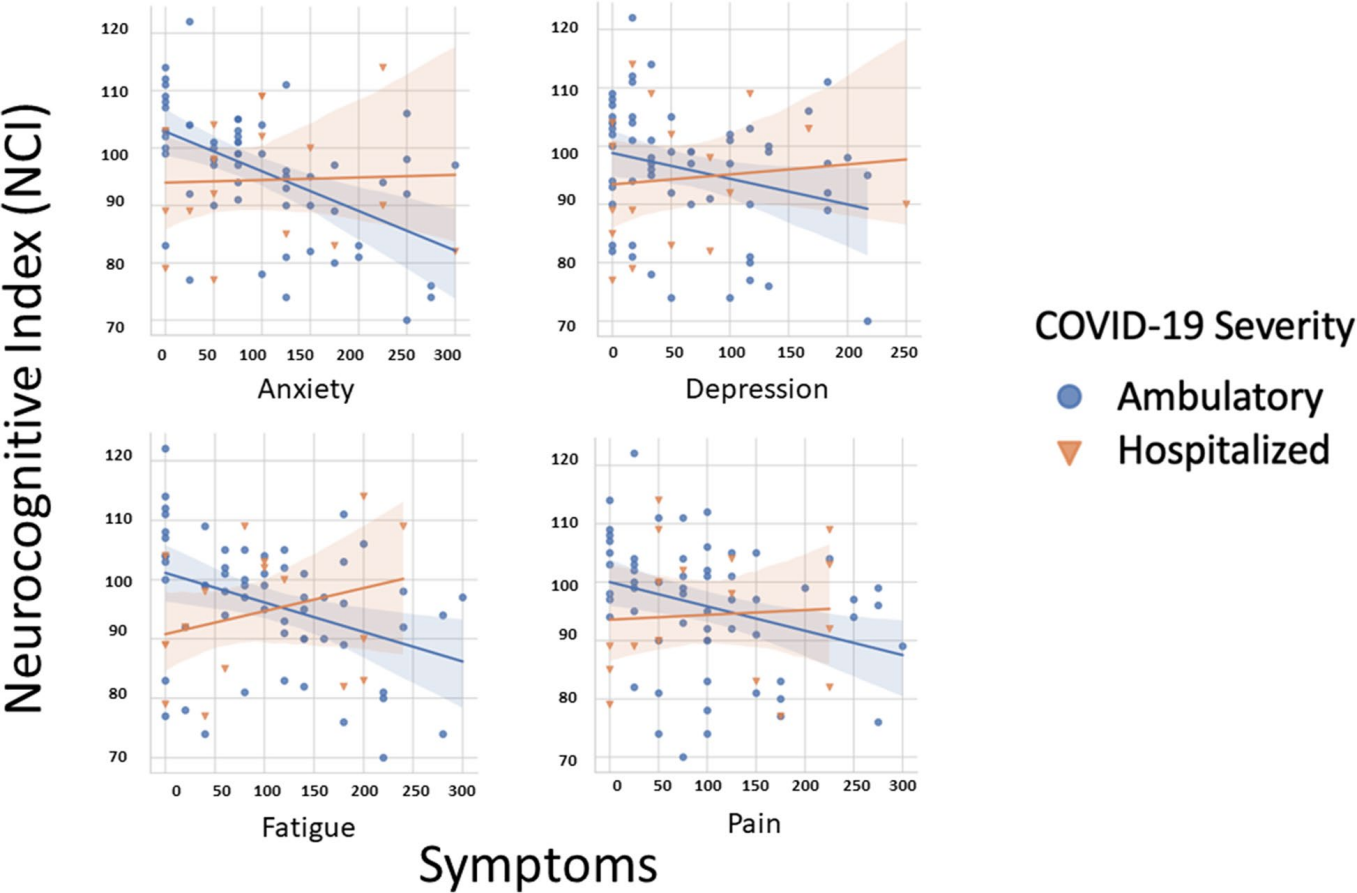
Higher subjective complaints of anxiety, depression, fatigue, and pain are associated with lower objective neurocognitive performance in ambulatory, but not hospitalized, patients

To determine whether subjective neuropsychological complaints were differentially associated with objective cognitive performance in the ambulatory and hospitalized groups, we analyzed the correlation between NPQ-45 domain scores and the overall NCI (Fig. 3). In ambulatory patients, lower NCI scores were associated with higher subjective complaints of attention (rho = -0.42, *p* = 0.001), memory (rho = -0.57, *p* < 0.001), anxiety (rho = -0.30, *p* = -0.02), depression (rho = -0.28, *p* = 0.03), fatigue (rho = -0.32, *p* = 0.01), and pain (rho = -0.376, *p* = 0.003). In contrast, within the hospitalized group, there were no significant correlations between NCI scores and subjective complaints of attention (rho = 0.31, *P* = 0.23), memory (rho = 0.34, *p* = 0.70), anxiety (rho = 0.22, *p* = 0.41), depression (rho = 0.37, *p* = 0.15), fatigue (rho = 0.34, *p* = 0.18), or pain (rho = 0.15, *p* = 0.57).

## Discussion

Neurocognitive complaints were common in patients recovering from COVID-19 in this series, regardless of disease severity; however, the rate of objective impairment was higher in hospitalized patients. These results emphasize the importance of assessing *both* subjective and objective complaints in determining prevalence of cognitive impairment in recovering patients and research participants. In ambulatory patients, neurocognitive performance was closely linked to depression, anxiety, fatigue, and pain. This was not the case in hospitalized patients. These findings suggest that the drivers of cognitive complaints likely differ in hospitalized versus ambulatory COVID-19 patients. This is important as cause informs treatment. Of particular interest, biopsychosocial factors appear to be a strong driver of cognitive complaints in recovering ambulatory patients. These are treatable factors and interventions targeting anxiety, depression, sleep, and pain may prove to be the most efficient and cost-effective treatment approach to avert disability in patients with mild manifestations of COVID-19 [24, 25]. Objective neurocognitive deficits were more common in hospitalized patients—a marker of higher overall health comorbidities and COVID-19 disease severity—with predominant deficits in memory and psychomotor speed. Contributors to focal cognitive deficits in these patients are emerging, representing an important area for future research.

Memory deficits following hospitalization for COVID-19 are increasingly recognized [9, 10, 14, 26]. Previously identified predictors of impairment include hypoxemic respiratory failure [9, 10], delirium [10, 27], and inflammatory markers [28]. Independent of COVID-19, hypoxia is a known risk factor for long-term deficits in memory [29, 30]. Hypoxic/ischemic brain injury is the most common finding on autopsy of patients that died from COVID-19 [31]. Given the presence of respiratory distress in 87% of our hospitalized sample, hypoxic/ischemic injury to hippocampal networks may contribute to the focal memory impairment that we observed. Effects of sustained hypoxemia may be exacerbated by vascular risk factors. Indeed, association between vascular risk factors and objective outcomes were observed in this series, and others [10, 32]. Contributions from aberrant acute and chronic inflammation are also expected [11, 32, 33], with possible unmasking or exacerbation of incipient neurodegenerative disease processes [32], or shared pro-inflammatory risk factors for both Alzheimer’s disease and severe COVID-19 [34]. Longitudinal cognitive tracking is essential to clarify the various ways in which these risk factors interact to compound memory impairment in recovering patients.

In contrast to memory impairment, slower psychomotor speed was associated with comorbid medical conditions, specifically diabetes and obesity. This suggests that a history of diabetes and obesity may contribute to slowing of psychomotor processing speed, independent of COVID-19. A strong association between diabetes and psychomotor speed is already well established [35, 36]. Processing speed impairment alone may not be sufficient for justifying extensive neurologic/ neuroimaging work-up in patients with comorbid diabetes unless it is associated with other focal neurologic signs.

**Fig. 4:**
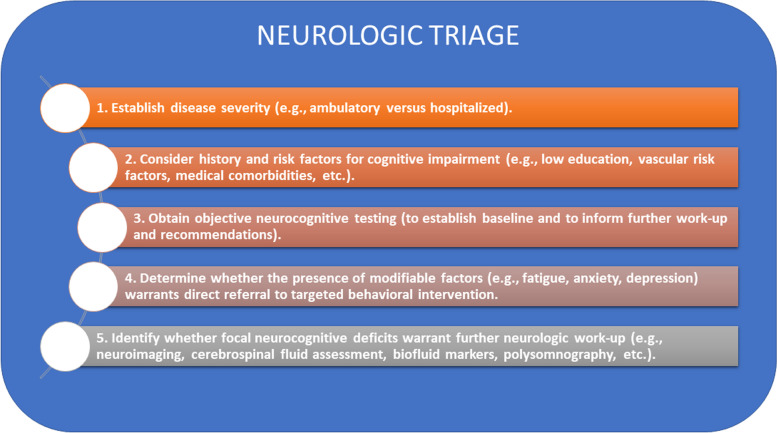
Flow diagram to guide decision-making when patients present with cognitive complaints in the post-acute recovery stage following SARS-CoV-2 infection

Asymptomatic or mild ambulatory patients performed, on average, within the normal range on objective cognitive measures. This is consistent with a prior study of ambulatory patients who presented to a Neuro-COVID-19 care clinic [3], which found no difference in cognitive test performance between patients who tested positive and negative for SARS-COV-2. Although the SARS-COV-2-positive patients scored lower than a demographic-matched US normative population on measures of attention and working memory, their mean scores were still within the average range [3]. Similar to our findings, they identified a moderate relationship between fatigue and cognitive performance [3]. This suggests that direct referral to behavioral programs designed to address chronic fatigue may offer the most efficient and cost-effective treatment approach for patients complaining of fatigue and ‘brain fog’ [37]. For patients with moderately-to-severely elevated anxiety and depression symptoms, referral for psychiatric evaluation should also be considered. Finally, the detection of focal neurocognitive deficits may warrant further neurological work-up to assess for structural contributors to dysfunction (e.g., neuroimaging to assess for stroke), biofluid markers of inflammation (e.g., cerebrospinal fluid assessment for leukocytosis, or serum measures of neurofilament light [33], or other issues (e.g., polysomnography to assess for occult sleep dysfunction), which may influence long-term prognosis or benefit from specific treatment (Fig. 4).

Study limitations include a small sample size of hospitalized patients. A larger proportion of the hospitalized patients in our sample completed outcome assessments compared with ambulatory patients. This suggests that remote computerized testing did not present a disproportionate access barrier for patients with more severe illness. Instances of delirium, seizures, and stroke were limited, precluding direct consideration of the contributions of these events to post-COVID19 subjective complaints and objective impairment. We relied on a 45-min computerized test battery, which eliminates exposure risk and is accessible to patients from remote locations; however, it requires a home desktop computer and computer literacy. Although this may have biased the sample towards a more socioeconomically advantaged and younger population, there were no differences in age, race, or ethnicity between those who did and did not complete the computerized outcome assessments. This suggests that if patients can electronically sign consent, computerized testing does not present an additional barrier. Given the cross-sectional nature of our study, we are unable to comment on the natural history and long-term risk of COVID-19 cognitive impairment. It will be essential to track cognitive progression at future time points to determine the rate and predictors of cognitive normalization versus decline.

## Conclusions

Our study highlights that objective neurocognitive screening procedures should be performed if neurologic manifestations of COVID-19 are suspected based on subjective complaints. The telemedicine-enabled computerized test platform that we utilized appears to be sensitive to disease severity and focal effects (i.e., visual memory impairment) and falls within the scope of level 2 harmonization approach as specified by the NeuroCOVID Neuropsychology Taskforce [38]. Objective neurocognitive testing can inform clinical decision points regarding the need for further imaging or neurologic work-up. In many patients with mild cases of infection and normal performance on objective cognitive measures, it may be appropriate to directly refer them to a multi-disciplinary behavioral rehabilitation program to target mood, anxiety, sleep, and pain symptoms. Finally, our findings highlight the importance of including objective neurocognitive performance measures as endpoints in clinical trials investigating the cognitive consequences of COVID-19.

## Supplementary Information

**Additional file 1: Supplementary Table 1.** Neurocognitive Test Domains.

**Additional file 2: Supplementary Table 2.** Sociodemographic and clinical characteristics of patients lost to follow-up.

**Additional file 3: Supplementary Table 3.** Objective Performance on neurocognitive test domains by group.

## Data Availability

The datasets used and/or analysed during the current study are available from the corresponding author on reasonable request.

## Abbreviations

Amb: Ambulatory
COVID-19: Coronavirus disease 2019
CRC: COVID-19 Risk Calculation Score
EVCI: Elixhauser-van Walraven Comorbidity Index
Hosp: Hospitalized
NCI: Neurocognitive Index
RT-PCR: Real-time reverse transcriptase polymerase-chain-reaction
SARS-CoV-2: Severe acute respiratory syndrome coronavirus 2
