# Supplementary material for: Neurocognitive screening in patients following SARS-CoV-2 infection: tools for triage
Source: BMC Neurol. 2022 Jul 30;22:285. doi: 10.1186/s12883-022-02817-9 (PMC9338515; doi:10.1186/s12883-022-02817-9)
Supplement: Supplementary file 1 [file 12883_2022_2817_MOESM1_ESM.docx]

Supplementary Table 1. Neurocognitive Test Domains.

| Domain Score | Description |
| --- | --- |
| Neurocognitive Index (NCI) | Composite of the six neurocognitive domains described below. |
| Verbal Memory (VerM) | 15 target words presented visually, one at a time every 2 seconds, followed immediately by yes/no recognition testing of 15 targets and 15 foils. 15 targets and 15 new foils are presented again after a distraction-filled delay. Correct hits and correct misses are totaled across the immediate and delayed trials. |
| Visual Memory (VisM) | 15 target geometric figures presented visually, one at a time every 2 seconds, followed immediately by yes/no recognition testing of 15 targets and 15 foils. 15 targets and 15 new foils are presented again after a distraction-filled delay. Correct hits and correct misses are totaled across the immediate and delayed trials. |
| Psychomotor Speed (PS) | Total of right and left taps (in 10 s) from the finger tapping test and total correct responses (in 120 s) from the symbol digit coding test. |
| Reaction Time (RT) | Average of the two complex reaction time scores from the Stroop test (from the congruent and incongruent trials). |
| Complex Attention (CA) | Sum of errors from the continuous performance test, shifting attention test, and the Stroop test. |
| Cognitive Flexibility (CF) | Number of correct responses on the shifting attention test minus the number of errors on the shifting attention test and Stroop test. |
