# Supplementary material for: Neurocognitive screening in patients following SARS-CoV-2 infection: tools for triage
Source: BMC Neurol. 2022 Jul 30;22:285. doi: 10.1186/s12883-022-02817-9 (PMC9338515; doi:10.1186/s12883-022-02817-9)
Supplement: Supplementary file 2 [file 12883_2022_2817_MOESM2_ESM.docx]

Supplementary Table 2. Sociodemographic and clinical characteristics of patients lost to follow-up.

|  | Completed outcome assessments (n=102) | Did not complete outcome assessments (n=47) | p-value |
| --- | --- | --- | --- |
| Age, mean (SD) | 52.55 (14.64) | 48.62 (16.55) | 0.15 |
| COVID-19 Severity Classification, n (%) | 0.007 |
| Ambulatory | 77 (76%) | 44 (94%) |  |
| Hospitalized | 25 (24%) | 3 (6%) |  |
| Sex, n (%) | 0.72 |
| Males | 42 (41%) | 21 (45%) |  |
| Females | 60 (59%) | 26 (55%) |  |
| Race, n (%) |  |  | 0.58 |
| Native American | 0 (0 %) | 0 (0%) |  |
| Asian | 5 (5%) | 1 (2%) |  |
| Native Hawaiian | 0 (0%) | 0 (0%) |  |
| Black | 9 (9%) | 2 (4%) |  |
| White | 87 (85%) | 43 (92%) |  |
| More than one race | 1 (1%) | 1 (2%) |  |
| Ethnicity, n (%) |  |  | 0.46 |
| Hispanic | 5 (5%) | 4 (9%) |  |
| Non-Hispanic | 97 (95%) | 43 (91%) |  |
