# Supplementary material for: Neurocognitive screening in patients following SARS-CoV-2 infection: tools for triage
Source: BMC Neurol. 2022 Jul 30;22:285. doi: 10.1186/s12883-022-02817-9 (PMC9338515; doi:10.1186/s12883-022-02817-9)
Supplement: Supplementary file 3 [file 12883_2022_2817_MOESM3_ESM.docx]

Supplementary Table 3. Objective Performance on neurocognitive test domains by group.

|  | Overall (N=102) | Ambulatory (N=76) | Hospitalized (N= 26) | p-value |
| --- | --- | --- | --- | --- |
| Group mean and variance by cognitive domain (age-adjusted based on normative reference group; standardized scale: mean=100, SD=15) |
| Neurocognitive Index, mean (SD) | 95.59 (11.14) | 95.93 (11.14) | 94.41 (11.39) | 0.623 |
| Verbal Memory, mean (SD) | 94.04 (18.27) | 95.79 (17.31) | 89.00 (20.32) | 0.110 |
| Visual Memory, mean (SD) | 99.85 (14.36) | 101.78 (12.62) | 93.83 (17.80) | 0.020* |
| Psychomotor Speed, mean (SD) | 94.03 (15.58) | 95.45 (14.31) | 89.23 (18.87) | 0.109 |
| Reaction Time, mean (SD) | 92.78 (11.65) | 93.85 (11.63) | 89.47 (11.32) | 0.136 |
| Complex Attention, mean (SD) | 101.68 (13.32) | 102.03 (13.09) | 100.58 (14.30) | 0.682 |
| Cognitive Flexibility, mean (SD) | 95.78 (15.48) | 96.75 (15.18) | 92.63 (16.41) | 0.314 |
| Group proportions of patients showing impaired performance (<9th percentile) by cognitive domain |
| Neurocognitive Index, n (%) |  | 0.453 |
| Within normal limits | 68 (88%) | 53 (90%) | 15 (83%) |  |
| Impaired | 9 (12%) | 6 (10%) | 3 (17%) |  |
| Verbal Memory, n (%) |  | 0.096 |
| Within normal limits | 78 (80%) | 61 (84%) | 17 (68%) |  |
| Impaired | 20 (20%) | 12 (16%) | 8 (32%) |  |
| Visual Memory, n (%) |  | <0.001* |
| Within normal limits | 86 (90%) | 70 (96%) | 16 (70%) |  |
| Impaired | 10 (10%) | 3 (4%) | 7 (30%) |  |
| Psychomotor Speed, n (%) |  | 0.008* |
| Within normal limits | 76 (89%) | 63 (85%) | 13 (59%) |  |
| Impaired | 20 (11%) | 11 (15%) | 9 (41%) |  |
| Reaction Time, n (%) |  | 0.245 |
| Within normal limits | 78 (89%) | 60 (91%) | 18 (82%) |  |
| Impaired | 10 (11%) | 6 (9%) | 4 (18%) |  |
| Complex Attention, n (%) |  | 0.524 |
| Within normal limits | 72 (89%) | 55 (90%) | 17 (85%) |  |
| Impaired | 9 (11%) | 6 (10%) | 3 (15%) |  |
| Cognitive Flexibility, n (%) |  | 0.456 |
| Within normal limits | 67 (81%) | 52 (83%) | 15 (75%) |  |
| Impaired | 16 (19%) | 11 (17%) | 5 (25%) |  |
